# Mining Next Generation Sequencing Data: How to Avoid “Treasure in, Error Out”

**DOI:** 10.4172/2153-0602.1000e119

**Published:** 2015-06-06

**Authors:** Zhihua Jiang

**Affiliations:** Department of Animal Sciences and Center for Reproductive Biology, Washington State University, USA

## Editorial

During the last ten years, next generation sequencing methods, technologies and platforms have revolutionized genomics and transcriptomics research fields and advanced their applications in agriculture and biomedicine [1–3]. To date, the Roche 454 GS FLX(+) system, Applied Biosystems SOLiD (supported oligonucleotide ligation and detection) and Ion Proton/PGM/Chef systems now owned by Life Technologies (Grand Island, NY); Solexa GA (Genome Analyzer)/HiSeq/MiSeq/NextSeq developed by Illumina (San Diego, CA); and PacBio RSII system made by Pacific Biosciences (Menlo Park, CA) present five major platforms in the market. They utilize different sequencing chemistries (e.g., sequencing by ligation vs. sequencing by synthesis); templates (e.g., single molecules vs. clusters amplified by emulsion or bridge PCR); product sizes (e.g., from 75 bp to 8,500 bp in length) and number of reads per run (e.g., from one million to 5,000 million) [2,3].

Both DNA and RNA sequencing projects have been largely carried out using these platforms, which require construction of relevant libraries. Depending on the project goals, genomic DNA sequencing can be classified into 1) whole genome sequencing/re-sequencing and 2) reduced genome complexity sequencing. However, transcriptome analysis methods can be classified in different ways. When each transcript is considered as a unit, transcriptome sequencing can be grouped into two categories: 1) end to end (5′ – 3′ end) sequencing and 2) either 5′ end or 3′ end sequencing only. Alternatively, transcriptome analysis can be conducted by sequencing specific RNA sub-populations, such as polyA+ RNA sequencing, polyA- RNA sequencing, microRNA sequencing, circular RNA sequencing, isoform sequencing or others [2].

Nevertheless, the original DNA and RNA molecules are manipulated during library preparation. Fragmentation with chemical treatment, enzyme digestion or physical shearing is often used, breaking DNA/RNA molecules into pieces. Enrichment is a process to develop libraries for specific sub-groups or targets/signatures of DNA/RNA for sequencing. Both 5′ and 3′ end adaptors are ligated to fragmented DNA/RNA molecules, forming “5′ adaptor – DNA/RNA inserts – 3′ end adaptor” sandwiches that fit into sequencing platforms. To date, direct RNA sequencing [4] is the only method that does not require PCR in any part of the process, while other methods use PCR in both library preparation and sequencing stages (such as emulsion and bridge amplification). Conversion of RNA molecules into complementary DNA molecules by transcriptase is an essential step in gene expression profiling. Size selection of library products not only helps remove unused adaptors or oligos, but it also enhances sequencing efficiency set up for each sequencing platform. In addition, end repair and tailing are also needed in some methods. Certainly, these manipulations can produce biases, noise or artifacts, depending on how libraries are constructed.

## Biases

It is well known that RNA-seq data analyses possesses three biases: fragmentation bias, length bias and transcriptome composition bias [5,6]. When RNA molecules are chemically cleaved or physically sheared, fragmentation bias results in fewer reads derived from both 5′ and 3′ ends of transcripts, because the process favors their internal sequences. Short transcripts should have less total reads in libraries as compared to long transcripts, causing a length bias even though long and short transcripts are expressed at similar levels. Transcriptome composition bias results when one or a few transcripts in a given sample are expressed at extremely high levels, thereby downplaying the number of reads collected for other transcripts [6]. Therefore, several methods have been proposed to correct these biases, thus providing unbiased estimates of gene expression through data normalization. As pointed out recently by Finotello and colleagues [6], however, some methods remain error prone.

Genotyping by sequencing leads to biases as well, particularly in number of reads per sample, number of sites sequenced per sample and number of reads per site. For example, Byrne and colleagues [7] reported that coefficients of variation ranged from 27% to 62% for number of reads per sample. The authors found that both barcode (F(31, 223) = 20.93, p,0.001) and barcode length (F(4, 250) = 29.89, p,0.001) had significant effects on read numbers per sample. In addition to variation in number of reads per library, Chen et al. [8] also observed variations in number of sites with reads mapped per library, ranging from 171,472 to 447,051 sites per sample. Based on the *Zea mays* genome reference, frequency of enzyme *ApeK*I recognition sites in the reference genome and size selection of 70 – 318 bp in library construction, Beissinger and colleagues [9] predicted that this process should yield reads for a total of 1,406,269 sites. Theoretically, they expected that the number of reads per site would follow a Poisson distribution with mean equal to the average coverage (~40 reads per site). However, in reality, 0 reads were recorded for 1,021,382 sites and the remaining sites had 1 – 95,014 read(s) per site.

## Noise

End-to-end sequencing of transcripts by RNA-seq is not cost-effective for conventional transcriptome analyses. As such, the community has developed more than 15 methods and technologies to carry out whole transcriptome tag/target sequencing [2]. These methods can produce noisy reads from unwanted products generated in library construction. For example, Ma and coworkers [10] compared different methods for target sequencing of PATs (polyA tags). Surprisingly, the authors reported that the percentage of reads mapped to the 3′untranslated regions accounted for 1.8% to 52.8% among 20 libraries. This means that the noisy read rate ranged from 47.2% to 98.2%. These results clearly indicate that noisy data can be overwhelmingly produced at some point during the library preparation process.

The SAGE (serial analysis of gene expression) technique was designed to catch one tag per transcript only [11]. However, this is not true in practice. When we studied the reactomes of porcine alveolar macrophages infected with porcine reproductive and respiratory syndrome virus (PRRSV), we found that noisy tags were abundant [12]. In addition to the 3′ most tag, for example, at least 10 other tags for pig interleukin 1, alpha (*IL1A*) were present in the library. Generally speaking, the 3′ most tag is expressed most abundantly, but the numbers of tags per million (TPM) for other tags decrease significantly depending on their distances from poly(A) end of the mRNA. Nevertheless, the results indicate that SAGE-based tools generate noisy data for transcriptome analysis. In fact, only the 3′ most cut site tag is the only tag that should be used in analysis. If other tags were wrongly chosen for analysis, expression patterns/trends of a given gene might be dramatically different.

## Artifacts

Library preparation often uses ligases, either T4 ligase for DNA ligation or T4 RNA ligase for RNA ligation. As discussed above, ligation adds adaptors/linkers to 5′ end and/or 3′ end of DNA/RNA inserts, allowing them to fit in specific sequencing platforms. In reality, not only 5′ adaptors – DNA/RNA inserts – 3′ adaptors, but other combinations within inserts or between adaptors and inserts can occur randomly, especially when DNA/cDNA molecules are digested with restriction enzymes. Our recent experience shows that even a 120 bp read can contain three digested fragments that are located on different chromosomes. For example, after “unchaining” of the ligated artifacts, 106 million reads were increased up to 132 million reads with ≥ 36 bp in length. As a consequence, the unique mapping rate was also improved from ~45% to ~65%. In my opinion, the community needs to pay more attention to the artifact issues that occur during the preparation of next generation sequencing libraries.

## Summary

Here I just listed a few examples about biases, noisy data and artifacts due to DNA/RNA manipulation during preparation of the next generation sequencing libraries. Therefore, bioinformaticians should get to know how the libraries are prepared before they can develop programs and software to handle these challenges sufficiently and carefully. In particular, we need to be cautious if we would develop tools to explore polyadenylation events using RNA-seq data, because the 3′ UTR reads are biased and incomplete. We would not collect reliable information if we simply use the tag counts derived from a SAGE library or its derivatives to determine alternative polyadenylation events or proximal to distal/distal to proximal site shifts. Furthermore, ignoring artifact reads is not cost-effect. Next generation sequencing technologies generate high quality reads since none of the platforms use Sanger sequencing. However, data analysis errors will occur if we do not pay attention to biases, noisy data and artifacts.

## References

[1] Jiang Z, Rokhsar DS, Harland RM (2009). Old can be new again: HAPPY whole genome sequencing, mapping and assembly. Int J Biol Sci.

[2] Jiang Z, Zhou X, Li R, Michal JJ, Zhang S (2015). Whole transcriptome analysis with sequencing: methods, challenges and potential solutions. Cell Mol Life Sci.

[3] Hodkinson BP, Grice EA (2015). Next-Generation Sequencing: A Review of Technologies and Tools for Wound Microbiome Research. Adv Wound Care (New Rochelle).

[4] Ozsolak F, Platt AR, Jones DR, Reifenberger JG, Sass LE (2009). Direct RNA sequencing. Nature.

[5] Wang Z, Gerstein M, Snyder M (2009). RNA-Seq: a revolutionary tool for transcriptomics. Nat Rev Genet.

[6] Finotello F, Di Camillo B (2015). Measuring differential gene expression with RNA-seq: challenges and strategies for data analysis. Brief Funct Genomics.

[7] Byrne S, Czaban A, Studer B, Panitz F, Bendixen C (2013). Genome wide allele frequency fingerprints (GWAFFs) of populations via genotyping by sequencing. PLoS One.

[8] Chen Q, Ma Y, Yang Y, Chen Z, Liao R (2013). Genotyping by genome reducing and sequencing for outbred animals. PLoS One.

[9] Beissinger TM, Hirsch CN, Sekhon RS, Foerster JM, Johnson JM (2013). Marker density and read depth for genotyping populations using genotyping-by-sequencing. Genetics.

[10] Ma L, Pati PK, Liu M, Li QQ, Hunt AG (2014). High throughput characterizations of poly (A) site choice in plants. Methods.

[11] Velculescu VE, Zhang L, Vogelstein B, Kinzler KW (1995). Serial analysis of gene expression. Science.

[12] Jiang Z, Zhou X, Michal JJ, Wu XL, Zhang L (2013). Reactomes of porcine alveolar macrophages infected with porcine reproductive and respiratory syndrome virus. PLoS One.

